# Supplementary material for: Longitudinal Analysis and Comparison of Six Serological Assays up to Eight Months Post-COVID-19 Diagnosis
Source: J Clin Med. 2021 Apr 21;10(9):1815. doi: 10.3390/jcm10091815 (PMC8122444; doi:10.3390/jcm10091815)
Supplement: Supplementary file 1 [file jcm-10-01815-s001.zip › jcm-1144056-supplementary.pdf]

Supplementary Materials:

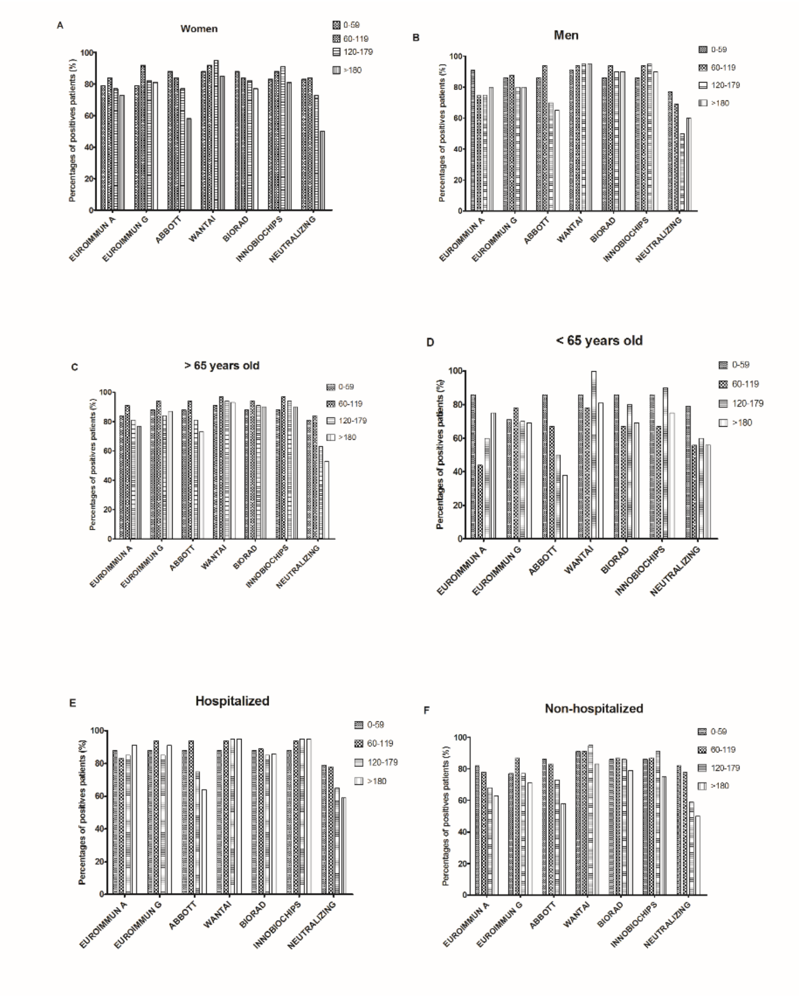

Figure S1. Percentage of SARS-CoV-2 positive patients in various serological assays by post-PCR period for women (A), men (B), people over 65 years of age (C), people under 65 years of age (D), people hospitalized for COVID-19 (E), and people not requiring hospitalization (F).

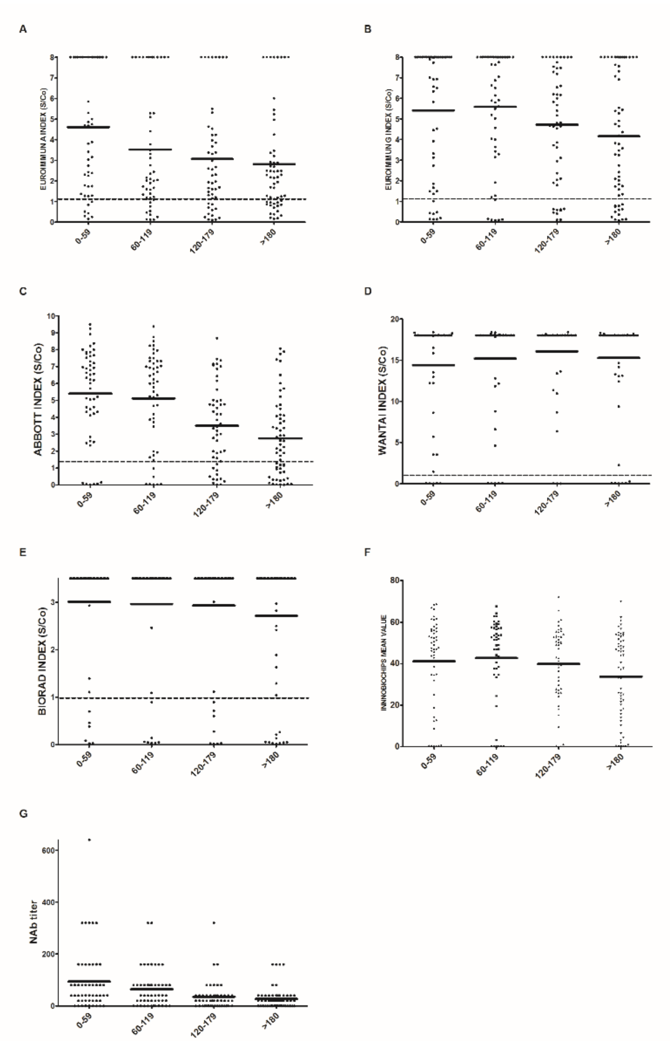

Figure S2. Raw data of the results of the 208 samples by assay according to the collection period after the first positive PCR.

Table S1. Characteristics of the serological assays.

	ABBOTT®	BIORAD®	EUROIMMUN®	WANTAI®	INNOBIOCHIPS®	NEUTRALIZING ASSAY
**Targeted viral antigen**	Nucleocapsid	Nucleocapsid	Spike 1	Receptor-binding domain (RBD)	Spike1, Spike 2, Nucleocapsid, Receptor-binding domain and N-terminal domain	Spike
**Immunoglobulins detected**	IgG	Total antibodies	IgA and IgG	Total antibodies	IgG	Neutralizing antibodies
**Formats**	CLIA Indirect antigen down	ELISA Double antigen bridging	ELISA Indirect antigen down	ELISA Double antigen bridging	Multiplex ELISA	Pseudovirus assay
**Positivity threshold**	≥ 1.4	≥ 1	≥ 1.1	≥ 1	Algorithm according to arbitrary units for each antigen	Any titer > 10

CLIA : Chemiluminescence Immunoassay; ELISA : Enzyme-linked immunosorbent assay.
